# Supplementary figures and images for: Tie2 inhibition disrupts TMEM doorway function and reduces dissemination in pancreatic ductal adenocarcinoma
Source: J Exp Clin Cancer Res. 2026 May 16;45:126. doi: 10.1186/s13046-026-03730-6 (PMC13196212; doi:10.1186/s13046-026-03730-6)

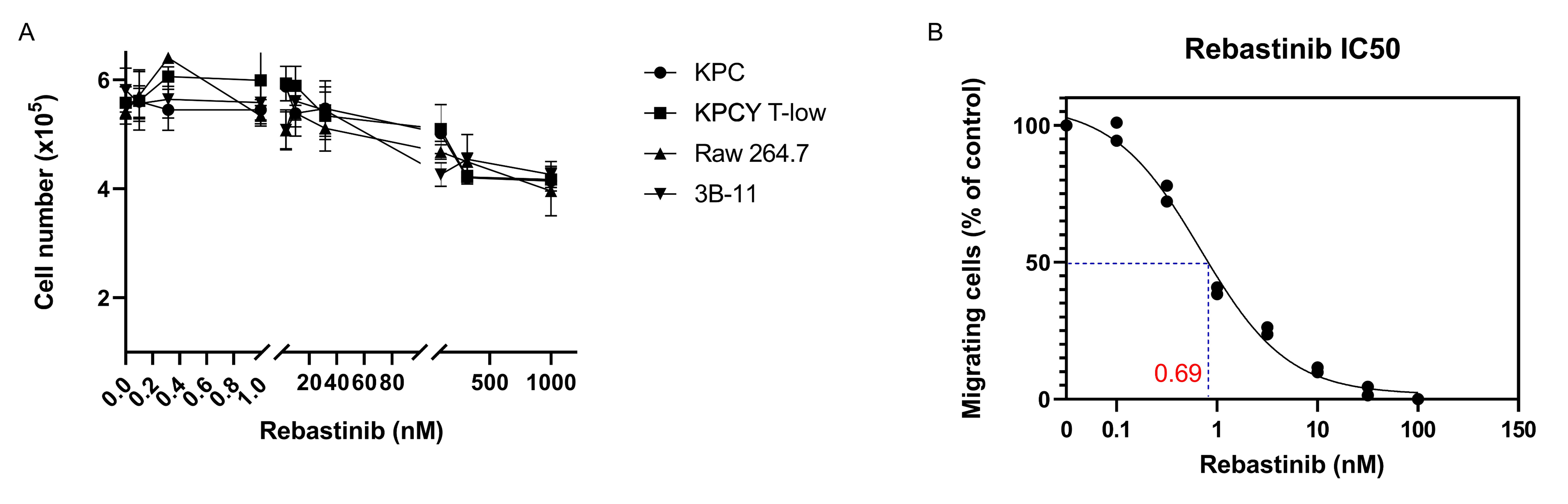

Supplement: Supplementary file 3 — Supplementary Material 3. [file 13046_2026_3730_MOESM3_ESM.tif]

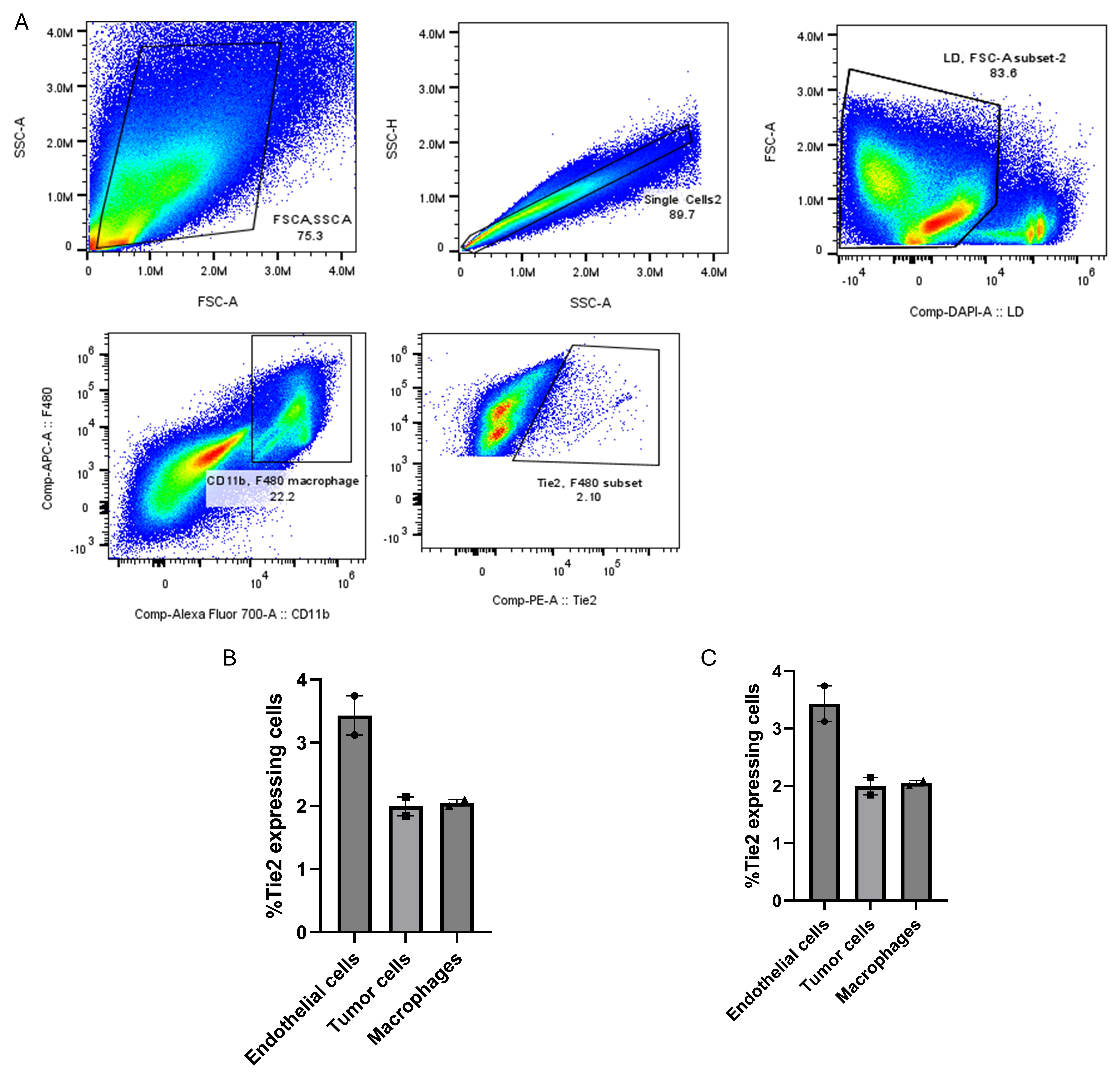

Supplement: Supplementary file 5 — Supplementary Material 5. [file 13046_2026_3730_MOESM5_ESM.tif]

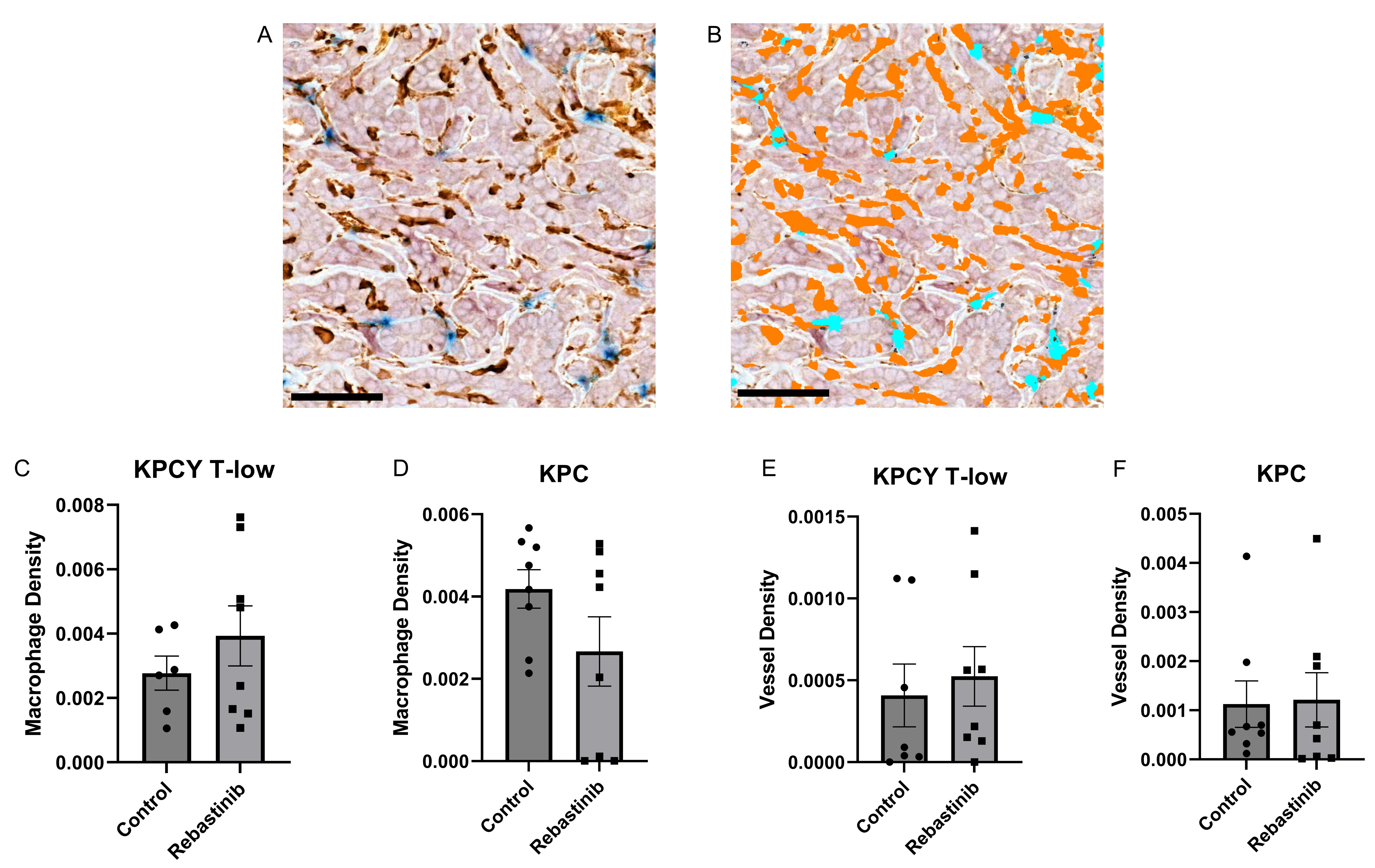

Supplement: Supplementary file 6 — Supplementary Material 6. [file 13046_2026_3730_MOESM6_ESM.tif]

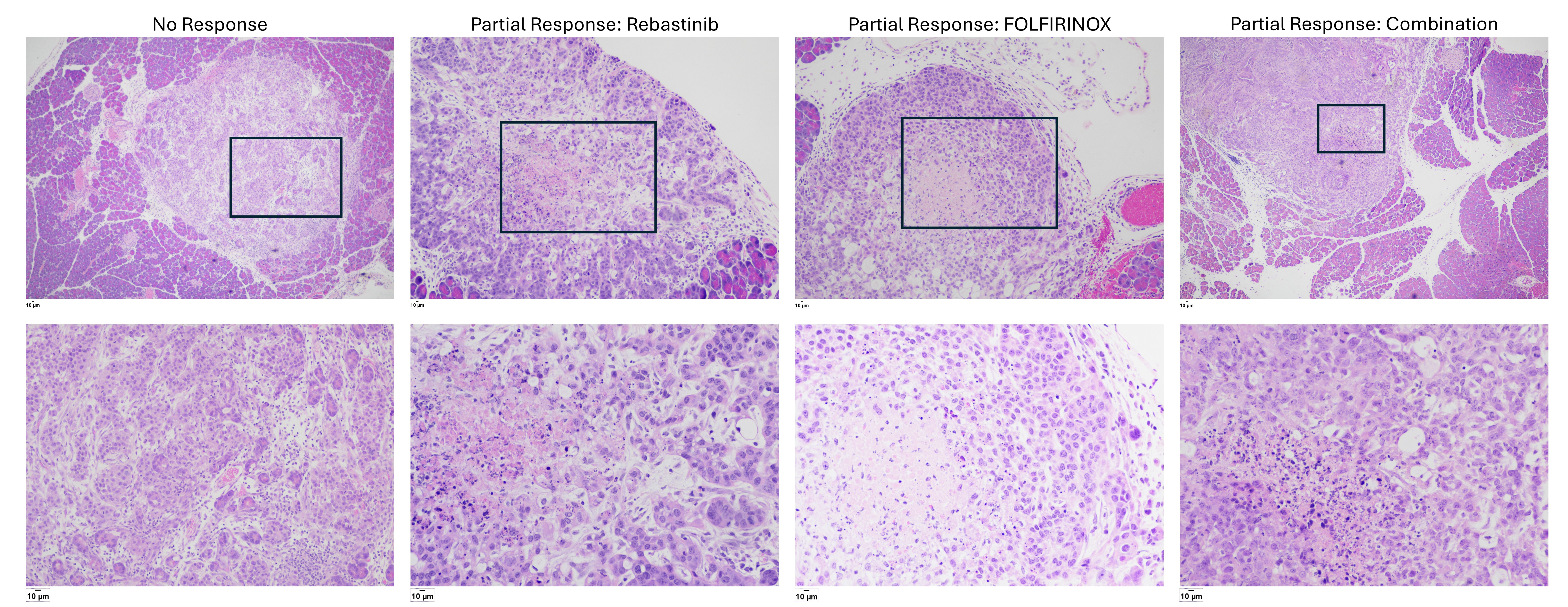

Supplement: Supplementary file 7 — Supplementary Material 7. [file 13046_2026_3730_MOESM7_ESM.tif]
